# Does Apoptosis Regulate the Function of Retinal Photoreceptors?

**Published:** 2012

**Authors:** Reginald Halaby

**Affiliations:** Department of Biology and Molecular Biology, Montclair State University, Montclair USA

**Keywords:** Apoptosis, Retinal photoreceptors, Pathophysiology

## Abstract

Apoptosis, or programmed cell death, is an integral component of developmental biology, embryology, and anatomy. All eukaryotic cells possess the molecular machinery necessary to execute apoptosis. However, dysregulated apoptosis in the form of too much or too little cell death results in diseases such as Alzheimer’s disease, autoimmune disorders, and cancer. It is postulated that apoptosis of the photoreceptors in the retina plays a vital role in mediating vision, and evidence is presented here to support this hypothesis. However, the precise mechanisms that regulate this cell death in photoreceptors have yet to be fully elucidated.

## INTRODUCTION

Apoptosis, or programmed cell death, comes from a Greek term meaning “the falling off of the leaves”. Apoptosis is also known as cell suicide and is a mechanism that is used in eukaryotic cells to regulate cell numbers. It can be considered as the opposite of mitosis. Apoptosis is a normal part of development and is required during limb formation, creation of the central nervous system, and wound healing. The suppression of apoptosis increases the susceptibility of an individual to malignancy, whereas uncontrolled apoptosis is associated with degenerative diseases. These include acquired immunodeficiency syndrome (AIDS) [1], cancer [2], Parkinson's disease [3], and Alzheimer's disease [4]. In the visual system of developing vertebrates, apoptosis preferentially eliminates neurons that form improper connections [5]. The neural retina gives rise to photoreceptors, retinal ganglion cells, and the optic nerve, and as many as 90% of newborn retinal ganglion cells die during rat retinal development [6,7]. In Xenopus, spatiotemporal elimination of retinal cells is a key factor in maturation [8]. In the chick, caspase-dependent apoptosis has been demonstrated in the retina, and the inhibition of caspases results in an enlargement of the ganglion cell layer [9]. Similarly, in mice, apoptotic factors are highly expressed in the early retina and down-regulated as development proceeds [10, 11], and the knockdown of caspases results in an overgrown retina [12]. However, the factors that are important for the regulation of caspases and other apoptotic factors in the eye are unknown.

Apoptosis is the main mechanism of cell loss in induced [13,14] or inherited retinal degeneration [15,16] in animal models, and it may represent the mechanism of cell death in many human retinal diseases. Excessive light may enhance the progression and severity of human age-related macular degeneration, as well as some forms of retinitis pigmentosa [17,18]. Likewise, several animal models with inherited retinal degeneration show an increased susceptibility to light damage compared with control animals [19]. Exposure to excessive levels of white light induces photoreceptor apoptosis, thus providing an excellent model to analyse degenerative photoreceptor loss [20].

It was shown that rod photoreceptors of mice lacking the c-Fos component of the transcription factor activator protein-1 (AP-1) (c-fos^−/−^) are resistant to light (5 klux for 2 hr) that induces apoptosis in wild-type (c-fos^+/+^) mice [21]. The basis for this protection and the role of c-Fos in light-induced rod apoptosis are currently unknown. Rods in retinas of c-fos^−/−^ mice are functional and, during a period of light exposure that is sufficient to induce apoptotic death of rods in wild-type mice, can absorb similar amounts of photons to the rods of wild-type mice [22]. However, light-induced apoptosis only occurs in c-fos^+/+^ mice. This suggests that an acute lack of c-Fos in light-induced apoptosis, rather than deficits in rod function induced by its absence, provides the basis for the resistance in c-fos^−/−^ mice. 

## Hypotheses and discussion

Apoptotic cell death in the retina was recently demonstrated in animal models of the hereditary human retinal dystrophy called retinitis pigmentosa [17,18]. Although recent evidence indicates that the proto-oncogene c-fos is a mediator of apoptosis, its precise role is unclear. In the retina, c-fos is physiologically expressed in a diurnal manner and is inducible by light [21]. White light (5 klux for 2 hr) induces apoptosis in rod photoreceptors in wild-type mice (c-fos^+/+^) within 24 hr, whereas the rods of c-fos knock-out mice (c-fos^−/−^) are protected [21]. The increased activity of the transcription factor activator protein-1 (AP-1) in retinas of light-exposed c-fos^+/+^ mice indicated the acute contribution of AP-1 in apoptosis induction. AP-1 activity increased during exposure and peaked approximately 6 hr thereafter, coinciding with the appearance of the major morphological signs of apoptosis [23].

The signalling of pro-apoptotic stimuli involves so-called private pathways: a multitude of different signal cascades, which may converge at the level of mitochondria [24,25]. The release of cytochrome c and/or an apoptosis-inducing factor [26,27], which is attributable to changes in the mitochondrial membrane, appears to represent a point of no return in apoptosis [28,29]. Their presence in the cytosol can trigger the common pathway of apoptosis, involving a tightly-regulated cascade of caspases [30-32]. Preliminary evidence indicates that light-induced photoreceptor apoptosis might involve the activation of cytochrome c-responsive caspases [33]. In contrast to c-fos^+/+^ mice, the morphology of mitochondria in c-fos^−/−^ mice appeared to be unaffected by light exposure [21]. It can be assumed that the morphological changes of mitochondria in c-fos^+/+^ mice reflect their involvement in the induction of apoptosis. Thus, in the absence of c-Fos, the private pathway that signals the stimulus excessive light appears to be interrupted before mitochondrial morphology is affected. 

## CONCLUSION

Strong evidence has accumulated for an acute and specific contribution of AP-1 containing c-Fos to the induction of light-induced apoptosis in rods. Although all of the collected evidence strongly supports this model [22], as yet undetected subtle changes of retinal development in c-fos^−/−^ mice might also contribute to an increased light tolerance. Further studies are warranted to gain a better understanding of the exact mechanisms by which apoptosis modulates the function of retinal photoreceptors. The data obtained from these studies should lead to novel and more effective treatments for retinal diseases, such as diabetic retinopathy and macular degeneration.

## DISCLOSURE

The authors report no conflicts of interest in this work.
